# Mobile health application for the treatment of urinary incontinence after radical prostatectomy: development and quality analysis

**DOI:** 10.1590/1980-220X-REEUSP-2024-0119en

**Published:** 2024-10-21

**Authors:** Fabrícia Eduarda Baia Estevam, Adriana Ferreira Machado, Cissa Azevedo, Lívia Cristina de Resende Izidoro, Filipe Maciel de Souza dos Anjos, Hugo Miranda de Oliveira, Sérgio Teixeira de Carvalho, Luciana Regina Ferreira da Mata

**Affiliations:** 1 Universidade Federal de Minas Gerais, Escola de Enfermagem, Belo Horizonte, MG, Brazil.; 2 Universidade Federal de São João del-Rei, Divinópolis, MG, Brazil.; 3 Universidade Federal de Jataí, Jataí, GO, Brazil.; 4 Universidade Federal de Goiás, Instituto de Informática, Goiânia, GO, Brazil.

**Keywords:** Nursing, Urinary Incontinence, Behavior Therapy, Mobile Applications, Medical Informatics, Technological Development and Innovation Projects, Enfermería, Incontinencia Urinaria, Terapia Conductista, Aplicaciones Móviles, Informática Médica, Proyectos de Desarrollo Tecnológico e Innovación

## Abstract

**Objective:**

To describe the development and quality analysis stages of a mobile health application for the treatment of urinary incontinence in men after radical prostatectomy.

**Method:**

A technological development study. Eight clinical experts and eight software development experts participated in quality assessment. Six characteristics and 22 subcharacteristics were assessed using an online form. Agreement rates above 70% were considered satisfactory.

**Results:**

The percentages of agreement of characteristics by clinical experts and developers were performance efficiency (90.5%), compatibility (100%) (both assessed only by the developers), functional suitability (78.5; 100%), usability (74.2; 82.7%), reliability (95.0; 82.3%) and security (87.6; 91.4%). Accessibility, user error protection, maturity and recoverability subcharacteristics showed agreement below 70%, which guided researchers to incorporate software improvements.

**Conclusion:**

The application presented satisfactory technical quality, configuring digital technological innovation that favors nursing care for men with urinary incontinence after radical prostatectomy.

## RESUMEN

**Objetivo:**

Describir las etapas de desarrollo y análisis de calidad de una aplicación móvil de salud para el tratamiento de la incontinencia urinaria en hombres postprostatectomía radical.

**Método:**

Estudio de desarrollo tecnológico. En la evaluación de calidad participaron ocho especialistas del área clínica y ocho en desarrollo de software. Se evaluaron seis características y 22 subcaracterísticas mediante un formulario en línea. Las tasas de acuerdo superiores al 70% se consideraron satisfactorias.

**Resultados:**

Los porcentajes de acuerdo sobre las características por parte de expertos clínicos y desarrolladores fueron eficiencia de rendimiento (90,5%), compatibilidad (100%) (ambos evaluados únicamente por los desarrolladores), adecuación funcional (78,5;100%), usabilidad (74,2;82,7%), confiabilidad (95,0;82,3%) y seguridad (87,6;91,4%). Las subcaracterísticas de accesibilidad, protección contra errores, madurez y recuperabilidad mostraron una concordancia inferior al 70%, lo que guió a los investigadores a incorporar mejoras de software.

**Conclusión:**

La aplicación presentó calidad técnica satisfactoria, representando una innovación tecnológica digital que favorece la atención de enfermería a hombres con incontinencia urinaria postprostatectomía radical.

## INTRODUCTION

In the context of male urinary incontinence (UI), mobile applications aimed exclusively at this audience are scarce, especially with regard to UI resulting from surgical treatment for prostate cancer^(1)^. UI affects approximately 60% of men after radical prostatectomy and negatively impacts quality of life^(2)^ from a social, emotional and sexual perspective^(3)^. Behavioral measures based on changes in lifestyle habits and pelvic floor muscle training are the first conservative treatment strategies^(2)^. The implementation of behavioral measures through a cognitive behavioral program becomes important, as it helps to increase adherence to treatment and, consequently, a greater chance of therapeutic success^(3)^.

Mobile health (mHealth) refers to the use of mobile technologies in healthcare, in order to improve communication between professionals and users as well as self-management of certain clinical conditions^(4,5)^. Technological advances linked to greater dissemination of digital resources in the market and accessibility by the general population have favored the insertion of these technologies in the health field, both in assistance and in care management^(4)^. In this context, several mHealth applications are available in online stores, with the aim of expanding therapeutic possibilities in different areas. However, scientific rigor in the structuring of content and the use of minimum software engineering quality requirements for developing these technologies are still incipient^(6)^.

Software engineering methodologies aim to establish quality requirements for mHealth applications^(7)^, with emphasis on the International Organization for Standardization/International Electrotechnical Commission (ISO/IEC) 25010:2011 System and software engineering – Systems and software Quality Requirements and Evaluation (SQuaRE)^(8)^, which defines parameters to specify, measure and assess software in terms of product quality and use. Therefore, the development of a technology with guaranteed quality requirements can significantly impact the achievement of better clinical results for users.

In a systematic search carried out on the Play Store^®^ and App Store^®^ platforms, with the aim of assessing the suitability and usefulness of mobile applications aimed at the conservative treatment of male UI, only three software programs were identified^(9)^. During assessment, restrictions were found regarding language, functionality and resources offered, especially regarding guidelines on how to perform treatment and the lack of technical support for users^(9)^. Thus, with a view to proposing a new digital technology aimed at self-care and improving men’s quality of life, this study aimed to describe development stages and quality analysis of an mHealth application for treatment of UI in men after radical prostatectomy.

## METHOD

### Study Design and Place

This is a technological development and assessment study that aims to build an mHealth application based on the unified process methodology^(7)^. The research was carried out simultaneously in two public higher education institutions in Brazil, one located in Minas Gerais and the other in Goiás. The study took place in two phases, development and product quality analysis^(8)^, from April 2021 to March 2022.

### Selection Criteria and Sample Definition

A sample of 16 experts was considered for the product quality analysis phase^(10,11)^, eight with experience in the clinical area related to UI and eight in the software development area. The survey of potential experts was carried out through snowball convenience sampling^(12)^. Members of research groups were asked to recommend professionals with experience in the areas, with a view to obtaining greater accuracy in assessment.

The criteria used to select experts were adapted from a scoring system for validity studies^(13)^. The following criteria were considered: having a thesis or dissertation on urinary dysfunctions or software development (5 points/title); nurses with specialization in stomatherapy, urology or pelvic floor dysfunctions and computer science professional expert in software development (2 points/title); participation in research groups or projects related to nursing care in voiding dysfunctions or software development (1 point); teaching experience in the subject of voiding dysfunctions or software development (1 point/year); authorship in at least three works related to voiding dysfunctions or software development published in journals in the last five years (0.5 points/work); participation in an assessment panel for works on urinary dysfunctions or software development (0.5 points/work)^(13)^. Professionals who achieved a minimum score of five points were included^(13)^. Those who did not have access to devices with the Android operating system were excluded, due to the impossibility of accessing the simulation environment.

The selected experts were invited via email. All participants received an instruction guide for accessing the software and a link to fill out the assessment form. Software development experts also received the technical report of the mHealth application with a description of the entire development flow, resources used and functionalities.

### Data Collection

The application development, guided by the unified process framework^(7)^, comprised five stages: communication (requirements gathering, definition of software objectives and functions, material resource selection); planning (task description, probable risk survey and work schedule definition); modeling (project refinement to understand the software needs); construction (project preparation); and delivery (software completion and product assessment).

In the communication stage, a descriptive, cross-sectional study was conducted to identify applications available for download on App Store^®^ and Play Store^®^ aimed at men with UI^(9)^. A total of 22 applications were analyzed using an adapted instrument, according to the scope of the application, literature used, in-app purchases, connectivity, advertisements, text search field, compatibility between devices, images/figures, videos and special characteristics. It was found that no application achieved the desired maximum score. The main gaps in clinical content and functionality identified in the available applications were succinct information about the exercises, lack of scientific theoretical basis for the content presented, and the absence of images or videos related to UI or the urinary system to illustrate how exercises should be performed or to help locate the pelvic muscles. Such gaps reinforced the need to develop new software with greater specificity for the target audience.

The subsequent stage, called planning, involved in software project construction, through remote meetings held every two weeks. The application development environments were defined, with the option of using free platforms and tools. Play Store^®^ was considered to make the application available, due to its scope^(14)^ and because it requires fewer financial resources for maintaining the software on the platform.

In the modeling stage, the mHealth application interface was structured based on the content and images of a printed educational technology entitled “Guidance manual on urinary incontinence after radical prostatectomy”^(8,15)^ and International Continence Society (ICS) clinical guidelines^(2)^. The content covered radical prostatectomy surgery and its complications, the definition of UI and its treatment, guidance on exercises for training the pelvic floor muscles, bladder diary and behavioral habits that help control UI. To define the application name, a non-probabilistic intentional sample was consulted, consisting of seven medical professionals and 12 nurses who work in urology and urinary dysfunctions. A form was made available on Google Forms^®^ that included the application’s objective, target audience and content to be made available on the technology as well as four name options for voting. At this stage, accessibility strategies^(16)^ were also established to be used in the application development, such as the use of neutral colors and easy-to-understand icons, simple language with short sentences, simplicity of commands to minimize access errors, animations for confirming commands between screens, audio guidance, sound signals and device vibration for alert messages.

The construction phase was based on mHealth application production with technology coding in computer language and storage on Play Store^®^. The interface was developed in the Javascript programming language with the React Native framework. The MySql database was used to store the information. The Figma tool was also considered for configuring and designing the screens, images, buttons, fonts and colors. The Expo^®^ platform was used to simulate the production environment during the software approval phase and to ensure that all researchers had access to the mHealth application on devices with the Android operating system.

**Figure 1:**
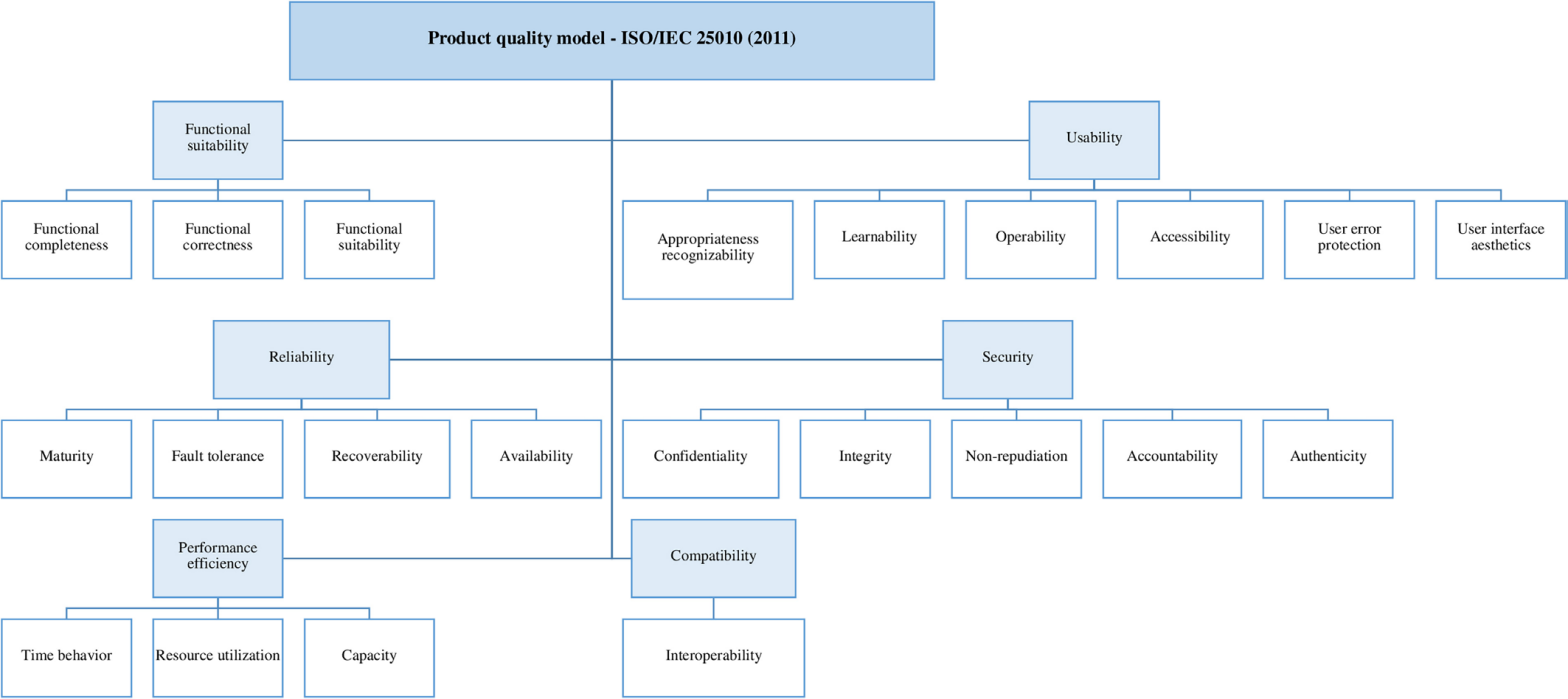
Characteristics and subcharacteristics of software product quality model used to assess the mHealth IUProst^®^ application.

Source: adapted from ISO/IEC 25010^(8)^.

The last stage, called delivery, involved navigation of the application by six researchers in order to check the functioning of the entire interface and the recording of errors. Subsequently, the corrected version of the software was made available on the Expo^®^ platform for product quality analysis by experts. Quality analysis of mHealth application followed the product quality model determinations^(8)^. This model assesses eight quality characteristics, such as functional suitability, performance efficiency, compatibility, usability, reliability, security, maintainability and portability. Each of these characteristics presents its respective subcharacteristics (n = 22), as shown in Figure 1.

Assessment forms were adapted from two instruments available in the literature^(10,17)^ and structured in Google Forms^®^. The clinical experts’ form included 24 items, which assessed four characteristics: functional suitability; usability; reliability; and security. The software development experts’ form included 33 items, which assessed six characteristics: functional suitability; performance efficiency; compatibility; usability; reliability; and security^(8)^. Maintainability and portability characteristics were disregarded for both groups of experts, since it was not possible to provide the software source code to participants^(8)^ (Figure 1).

For each sub-characteristic assessed (Figure 1), a classification/score was assigned: agree (1 point); disagree (zero points); not applicable (zero points). For “disagree” answers, experts could justify and suggest modifications in the comments field. A maximum period of 30 days was established for participants to respond. Researchers could be contacted via WhatsApp^®^, email or phone calls to solve possible technical problems with accessing the application.

### Data Analysis and Treatment

The data were transcribed into Microsoft Excel^®^ spreadsheets, and the percentage values of quality characteristics were obtained from the adapted formula: Vc = ∑ Vsca /(a + d + na – na) × 100^(16,17)^, where: measured value of the characteristic (Vc) is equal to the sum of subcharacteristics values with answers agree (∑Vsca), divided by the sum of answers agree (a), disagree (d) and not applicable (na), subtracted by the answers not applicable (na), multiplied by 100.

To calculate the mean and standard deviation of subcharacteristic values, the answer “not applicable” was excluded, as it referred to questions that experts were not able to assess, either due to lack of technical resources or specific knowledge. This answer did not score points and, therefore, did not compromise the assessment^(17)^.

For the analysis of results, the characteristics that obtained an agreement percentage higher than 70% were considered satisfactory, according to the assessment scale for subcharacteristics^(10,18)^, being 25% (poor), 50% (average), 75% (good) and 100% (excellent). All suggestions made by experts in the “comments” field were analyzed by the researchers regarding their relevance and feasibility to proceed with the software technical and structural adjustments. Suggestions that implied financial expenses and execution in a time frame that was not applicable to the established schedule were considered for future updates.

### Ethical Aspects

The research was approved by the *Universidade Federal de Minas Gerais* Research Ethics Committee, under Opinion 4,864,981 and CAAE (*Certificado de Apresentação para Apreciação* Ética – Certificate of Presentation for Ethical Consideration) 41736921.5.0000.5149, in accordance with recommendations of Resolution 466/12 of the Brazilian National Health Council. The volunteers who participated in the scenes of the videos that make up the application signed the Image Use Authorization Form. The video narrator is one of the main researchers, who signed the Voice Use Authorization Form. The Informed Consent Form (ICF) was also prepared and available in the application for each user to authorize the use of clinical data. The principles established in the General Data Protection Law were also considered during navigation in the application, in order to guarantee the integrity and privacy of each user’s data.

After assessing product quality, the application was approved and made available for download on Play Store^®^. IUProst^®^ registration was approved by the Brazilian National Institute of Industrial Property (INPI – *Instituto Nacional da Propriedade Industrial*), with a validity of 50 years from January 1^st^ following March 10^th^, 2022, in accordance with Article 2 of Law 9,609 of February 19^th^, 1998, Process BR512022001279-0.

## RESULTS

**Figure 2:**
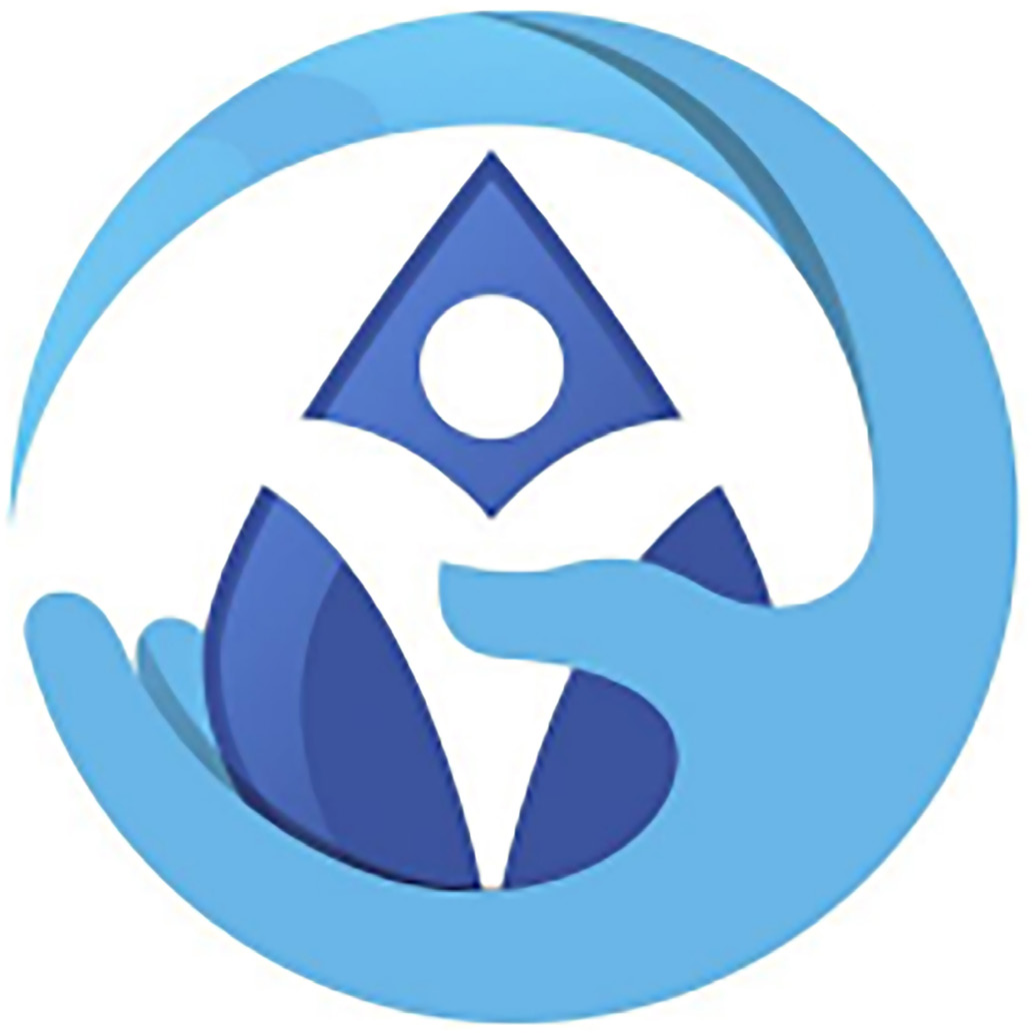
IUProst^®^ application logo (2022).

The name chosen for the application was “IUProst^®^”, which represents the combination of “IU”, meaning *incontinência urinária* (urinary incontinence), and “Prost”, as the root of the words prostate and prostatectomy. The logo characteristics a hand in light blue, which expresses the sense of help, protection and care; a drop in the center in dark blue, which refers to urinary loss; and an element in the center of the drop in white, which represents the man (Figure 2).

For initial access to the application, four opening screens were created with brief information about the application’s objective, ICF, registration, login and customization. The user customization screen refers to the insertion of clinical data and the completion of two clinical instruments: International Consultation on Incontinence Questionnaire - Short Form (ICIQ-SF)^(19)^; and Urinary Incontinence Scale After Radical Prostatectomy (UISRP)^(20)^.

Users are then directed to a main menu that includes eight screens. On the “About IUProst^®^” screen, there is general information about the application with data from the research group and references that support the content presented.

The “My exercises” screen was developed to guide users on how to train their pelvic floor muscles. The proposed exercises were organized into eight stages, to be performed over eight weeks (two months), in the morning, afternoon and evening. To facilitate understanding of how to perform the exercises, users can access brief explanations in text, audio and video format.

The “My bladder diary” screen shows the calculation of the recommended amount of fluids for daily intake^(21)^, a space to record the type, volume and time of fluids ingested, frequency of eliminations and urinary losses, frequency of changing liners, absorbent pads or diapers.

The “My treatment” screen contains information regarding the male urinary system anatomy, what is radical prostatectomy surgery, pelvic floor muscle recognition as well as tips on lifestyle habits that help improve UI. Interviews conducted with two users with UI after radical prostatectomy and a family member with reports of experiences and challenges faced in the face of the initial diagnosis and post-surgical treatment are available on a screen called “Patient experience”.

In “My achievements”, users monitor their performance in treatment through a graph that represents the execution of exercises in the last seven days and the daily consumption of liquids. The “Talk to a professional” screen enables contact between users and nursing professionals with experience in the area of urology via email, WhatsApp^®^ and Instagram^®^. The public can therefore clarify doubts and access information related to prevention, treatment and healthy lifestyle habits.

**Figure 3:**
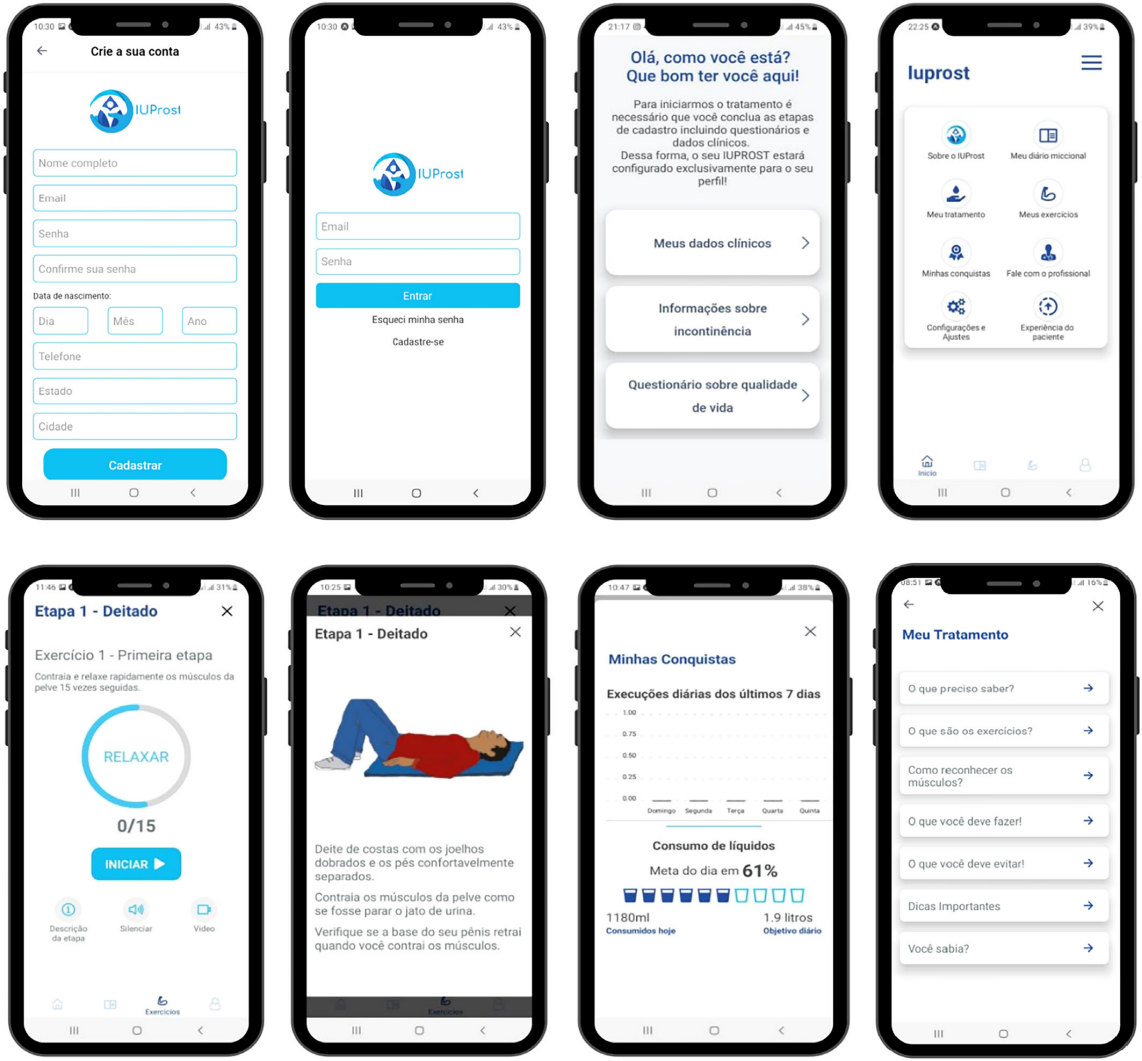
– IUProst^®^ application main menu screens. Belo Horizonte, MG, Brazil (2022).

In “Settings and adjustments”, users set the application according to their preferences, with schedules and sound alerts to remind them to perform pelvic floor muscle training exercises. Figure 3 shows some screens that make up IUProst^®^.

As for product quality assessment^(8)^, 30 evaluators were invited to participate in the research: 15 nursing professors and/or experts in the area and 15 software developers. Of these, only eight nurses and eight experts in software development responded to the invitation in advance. Of the eight clinical experts, the mean age was 44.6 years (± 6.1) and the mean time since graduation was 19.7 years (± 6.0). All of them have teaching experience in the subject, and 87.5% work in clinical practice. As for the eight experts in software development, the mean age was 36.7 years (± 8.9), and the mean time since graduation was 8.8 years (± 9.1). Most developers (75.0%) also have teaching experience in information technology, and 87.5% have practical experience in the area. Regarding geographic location, participants lived in the Central-West (n = 09), Northeast (n = 03), Southeast (n = 03) and South (n = 01) regions. According to the selection criteria^(13)^, clinical experts obtained a minimum score of 21.0 and a maximum of 42.5 points, whereas software development experts obtained a minimum score of 12.0 and a maximum of 54.0 points.

All characteristics analyzed regarding product quality presented indexes above 70%. In the analysis of the 22 subcategories, two obtained weak agreement (accessibility and user error protection). Two other subcategories presented regular agreement (maturity and recoverability). Table 1 describes the agreement indexes between clinical experts and software development experts. It is worth noting that clinical experts did not assess performance efficiency, compatibility, non-repudiation and accountability characteristics and subcharacteristics, as these are topics more specific to the area of computer science.

**Table 1:** Agreement index in product quality assessment between clinical experts and software developers – Belo Horizonte, MG, Brazil, 2022.

Characteristic and subcharacteristics	Software developers mean (SD)	Clinical experts mean (SD)
**Functional suitability**	**100.00 (0.0)**	**78.5 (7.1)**
Functional completeness	100.0 (0.0)	85.7 (0.0)
Functional correctness	100.0 (0.0)	78.5 (10.1)
Functional suitability	100.0 (0.0)	71.4 (0.0)
**Performance efficiency**	**90.5 (8.4)**	**–**
Time behavior	87.5 (0.0)	**–**
Resource utilization	100.0 (0.0)	**–**
Capacity	84.0 (21.9)	**–**
**Compatibility**	**100.0 (0.0)**	**–**
Interoperability	100.0 (0.0)	**–**
**Usability**	**82.7 (18.9)**	**74.2 (24.0)**
Appropriateness recognizability	96.8 (6.2)	92.2 (9.0)
Learnability	91.6 (14.4)	85.7 (0.0)
Operability	86.6 (1.3)	77.3 (8.4)
Accessibility	50.0 (0.0)	40.0 (0.0)
User error protection	71.4 (0.0)	50.0 (0.0)
User interface aesthetics	100.0 (0.0)	100.0 (0.0)
**Reliability**	**82.3 (20.5)**	**95.0 (10.0)**
Maturity	62.5 (0.0)	80.0 (0.0)
Fault tolerance	**100.0 (0.0)**	**100.0 (0.0)**
Recoverability	66.6 (0.0)	100.0 (0.0)
Availability	100.0 (0.0)	100.0 (0.0)
**Security**	**91.4 (9.3)**	**87.6 (5.4)**
Confidentiality	100.0 (0.0)	85.7 (0.0)
Integrity	93.7 (8.8)	93.7 (8.8)
Non-repudiation	100.0 (0.0)	**–**
Accountability	83.3 (0.0)	**–**
Authenticity	80.0 (0.0)	83.3 (0.0)

Note: SD – standard deviation.

Chart 1 presents experts’ comments that led to technical and structural improvements to the software.

**Chart 1:** Suggestions from clinical experts and software developers addressed in version 1 of the IUProst^®^ application – Belo Horizonte, MG, Brazil, 2022 (n = 16).

Quality characteristic	Software developers	Clinical experts
**Functional suitability**	– Correct progress evolution during the release of ingested liquids, urine volume and exercises performed (Expert 8). – Correct activation of the audio of one of the videos (Experts 1 and 2).	– Specify changes in lifestyle habits according to the patient’s complaint (Expert 2). – Add information on how to relax the pelvic muscles (Expert 2).
**Performance efficiency**	– Inform the database storage capacity (Expert 2). – Improve application loading time (Expert 4).	(Not applicable to this group of experts).
**Compatibility**	No comments/suggestions.	(Not applicable to this group of experts).
**Usability**	– Insert single answer fields, avoiding open-ended fields (Expert 3). – Insert “combos” in state and city registration (Expert 7). – Modify form fields to prevent the insertion of data with absurd and invalid values (Expert 6). – Include a screen with an explanation of the step in the “My exercises” section (Expert 8). – Insert the option to increase the font size of the texts (Expert 7).	– Include in clinical data the option to deselect previous diseases (Expert 7). – Include the option of no previous diseases (Expert 3). – Allow more than one alternative to be filled in for the first question of the quality of life questionnaire (Expert 4). – Provide accessibility support for people with disabilities, such as verbal commands for questionnaire questions and information about UI (Expert 8). – Adjust the language used in the application to make it more accessible to users (Expert 1). – Include mandatory steps or list the treatment steps so that users know which one to start with (Expert 3).
**Reliability**	– Insert progress record after data entries (Expert 1).	No comments/suggestions.
**Security**	– Insert password strength assessment (Expert 2). – Include a minimum number of characters for creating passwords (Expert 1).	– Insert password recovery mechanism (Expert 4). – Insert the option “automatically log in/save password on this device” (Expert 4).

## DISCUSSION

IUProst^®^ was developed as an mHealth technology capable of assisting healthcare professionals in monitoring and monitoring conservative treatment for UI after radical prostatectomy. This technological tool stands out for including specific content for the male audience regarding clinical guidance on UI, with a view to increasing adherence to treatment through interactive, dynamic strategies that are adjustable to users’ routine. The technology is also expected to strengthen the user-professional relationship by enabling communication channels and promoting the capacity for care self-management.

Health-related information and communication technologies are a set of tools that contribute to improving health information and diagnostic support. They can provide ease in the transmission of information through digital means, overcoming obstacles such as distance, expanding attention and care coverage as well as reducing costs^(22,23)^. In this context, mHealth applications have become relevant for healthcare, as they are tools that optimize and simplify the therapeutic process, in addition to contributing to expanding access to information and greater user engagement in treatment^(24)^. With the advancement of technological resources, it is expected that these tools will be gradually incorporated into clinical practice, with a view to assisting in the treatment and rehabilitation of certain clinical conditions^(5)^ as well as promoting health education^(25)^.

In the context of UI, in Brazil, most applications present reduced quality and limited functionalities^(9)^, mainly due to the lack of information based on reliable scientific bases^(26)^. In this regard, IUProst^®^ was developed based on the main ICS clinical guidelines^(2)^, structured according to the content of a cognitive behavioral program for the treatment of UI after radical prostatectomy with proven clinical effectiveness in clinical trials^(8,15)^. Furthermore, the application takes into account users’ unique characteristics by including characteristics that facilitate the monitoring of their clinical progress and customization of commands, according to their preferences and usage needs.

Researchers^(26)^ state that despite the large number of applications developed to support patients with UI, there are still few applications focused on patient needs as a way to promote better experiences and results. Conservative treatment, as the first therapeutic choice for UI, includes training the pelvic floor muscles in association with changes in lifestyle habits^(2)^. Since it involves human behavior, it requires strategies and tools that promote reinforcement of information during the therapeutic process, in order to increase adherence to treatment and clinical effectiveness. Therefore, it is essential to use valid and quality technologies that assist professionals and users in this purpose of behavioral change^(27)^.

Analyzing the quality of mHealth applications is essential for proposing improvements that favor navigability, digital accessibility and, consequently, greater adoption of technology^(27)^. The effective quality of a software is considered when the tool has elements that make it useful and provide measurable value, in different scenarios, with a focus on developers and users^(28,29)^. Thus, it is possible to propose a product that meets the target audience’s needs^(2)^. From this perspective, IUProst^®^ was assessed by two groups of experts with different expertise, which allowed greater robustness to the quality analysis process^(8)^.

IUProst^®^ obtained satisfactory agreement rates for all characteristics assessed^(8)^, which favors its recommendation as a technological tool allied to treatment, although it does not replace professional and in-person care. Functional suitability characteristic obtained 100% agreement among developers. However, among clinical experts, this characteristic showed an agreement of 78.5%. This result may be associated with suggestions for improving the clinical content of the application, such as specifying changes in lifestyle habits according to patient complaints and adding information on how to perform pelvic muscle relaxation.

As for information about pelvic muscle relaxation, it is worth noting that, in the “My treatment” tab, users have access to guidance on how to recognize their muscles and how to correctly relax and contract these muscles, in addition to the explanatory videos present in each training week. Therefore, the low agreement of functional suitability characteristic among clinical experts may be associated with the fact that some evaluators did not access all the resources available in the application, which resulted in a lower than expected result in the assessment of this characteristic.

Regarding reliability, two subcharacteristics obtained regular agreement by developers, such as maturity (62.5%) and recoverability (66.6%). Maturity refers to technical failures, and recoverability refers to the ability to recover data entered after an error message^(8)^. In fact, one evaluator reported that there was no record of progress in the exercise stage. However, to resolve this demand, a correction was made to the flow of recording exercise progress records, without subsequent losses.

Although usability characteristic obtained satisfactory levels of agreement between the two specialties, accessibility sub-characteristic showed weak agreement between clinical experts and developers. Accessibility concerns the support of people with disabilities, and, based on the results obtained, it is considered that this requirement requires improvements in IUProst^®^. To improve this requirement, clinical experts suggested the inclusion of verbal commands on the screen that includes the user assessment instruments. However, it is important to highlight that auditory accessibility was not planned by the developers in this first version of the software. On the other hand, it is worth highlighting the inclusion of audio and video on the screens related to the execution of muscle training exercises, which favors the use of the application by men with visual impairments.

Another usability sub-characteristic that showed weak agreement among clinical experts was user error protection. In this requirement, the objective was to investigate whether there is information for users in cases of invalid data entry^(8)^. To this end, it was proposed to insert unique answer fields that replaced all open-ended answer fields.

In performance efficiency assessment, which was carried out only by expert developers, time behavior, resource utilization and capacity subcharacteristics obtained good, excellent and good agreement rates, respectively. This result can be justified by the fact that IUProst^®^ has an exclusive server for data storage. The use of this server also contributes to the construction of a clinical database, which supports carrying out future epidemiological research as well as implementation of dashboards for clinical monitoring of users by health professionals.

Although IUProst^®^ was developed to support the treatment of men with UI after radical prostatectomy, the software can also be useful in professionals’ educational process. In addition to the exercise protocol, the cognitive behavioral program contains guidance on the main lifestyle habits, as well as explanations on anatomy and physiology, and teaching how to properly fill out the bladder diary. This diary refers to a low-cost tool recommended by ICS^(2)^, which allows characterizing individuals’ daily voiding habits and proposing better interventions for urinary elimination rehabilitation.

The limitations of this study include incompatibility of application with other operating systems, which makes it difficult for experts who do not have Android mobile devices to assess the software as well as failure to assess the quality of use by men who experience the health condition. Therefore, the research team’s short-term goals are to make IUProst^®^ available on the iOS operating system and assess it by the target audience through clinical effectiveness analysis studies. The research team will also move forward with the software redesign, in accordance with good practices for digital health literacy^(30)^, with a view to including new elements that facilitate navigation, understanding and clinical use of health information by users.

## CONCLUSION

IUProst^®^ development met the requirements set forth in the communication, planning, modeling, construction and delivery stages. It was found that all the quality characteristics assessed in IUProst^®^ were satisfactory, which demonstrates that the software presents technical and clinical quality to meet the needs of men with UI after surgical treatment for prostate cancer.

IUProst^®^ is a technological innovation in health that favors the role of nurses in care for men with UI after radical prostatectomy, based on valid information about male UI. The technology allows users to access guidance on changing lifestyle habits and performing exercises to strengthen the pelvic floor muscles, remotely and at any time, via a smartphone.
