# Supplementary material for: Mobile health application for the treatment of urinary incontinence after radical prostatectomy: development and quality analysis
Source: Rev Esc Enferm USP. 2024 Oct 21;58:e20240119. doi: 10.1590/1980-220X-REEUSP-2024-0119en (PMC11499247; doi:10.1590/1980-220X-REEUSP-2024-0119en)
Supplement: Translation

# Translation: Aplicativo mobile health para tratamento da incontinência urinária pós-prostatectomia radical: desenvolvimento e análise de qualidade

**DOI:** 10.1590/1980-220X-REEUSP-2024-0119pt

**Authors:** Fabrícia Eduarda Baia Estevam, Adriana Ferreira Machado, Cissa Azevedo, Lívia Cristina de Resende Izidoro, Filipe Maciel de Souza dos Anjos, Hugo Miranda de Oliveira, Sérgio Teixeira de Carvalho, Luciana Regina Ferreira da Mata

**Affiliations:** 1 Universidade Federal de Minas Gerais, Escola de Enfermagem, Belo Horizonte, MG, Brasil.; 2 Universidade Federal de São João del-Rei, Divinópolis, MG, Brasil.; 3 Universidade Federal de Jataí, Jataí, GO, Brasil.; 4 Universidade Federal de Goiás, Instituto de Informática, Goiânia, GO, Brasil.

**Keywords:** Enfermagem, Incontinência Urinária, Terapia Comportamental, Aplicativos Móveis, Informática Médica, Projetos de Desenvolvimento Tecnológico e Inovação

## Abstract

**Objetivo:**

Descrever as etapas de desenvolvimento e análise de qualidade de um aplicativo *mobile health* para tratamento da incontinência urinária em homens pós-prostatectomia radical.

**Método:**

Estudo de desenvolvimento tecnológico. Participaram da avaliação de qualidade oito especialistas da área clínica e oito em desenvolvimento de *software*. Foram avaliadas seis características e 22 subcaracterísticas por meio de formulário *online*. Índices de concordância acima de 70% foram considerados satisfatórios.

**Resultados:**

Os percentuais de concordância das características pelos especialistas da área clínica e desenvolvedores foram eficiência de desempenho (90,5%), compatibilidade (100%) (ambas avaliadas somente pelos desenvolvedores), adequação funcional (78,5;100%), usabilidade (74,2;82,7%), confiabilidade (95,0;82,3%) e segurança (87,6;91,4%). As subcaracterísticas acessibilidade, proteção contra erros, maturidade e recuperabilidade apresentaram concordância abaixo de 70%, o que orientou os pesquisadores para a incorporação de melhorias do *software.*

**Conclusão:**

O aplicativo apresentou qualidade técnica satisfatória, configurando-se inovação tecnológica digital que favorece o cuidado de enfermagem a homens com incontinência urinária pós-prostatectomia radical.

## INTRODUÇÃO

No âmbito da incontinência urinária (IU) masculina, aplicativos móveis direcionados exclusivamente para esse público são escassos, principalmente no que tange à IU decorrente do tratamento cirúrgico para o câncer de próstata^(1)^. A IU acomete cerca de 60% dos homens pós-prostatectomia radical e impacta negativamente a qualidade de vida^(2)^ na perspectiva social, emocional e sexual^(3)^. Medidas comportamentais pautadas em mudança nos hábitos de vida e treinamento dos músculos do assoalho pélvico são as primeiras estratégias de tratamento conservador^(2)^. A implementação das medidas comportamentais por meio de programa cognitivo comportamental torna-se importante, pois auxilia no aumento da adesão ao tratamento e, consequentemente, maior chance de sucesso terapêutico^(3)^.

*Mobile health* (*mHealth*) refere-se ao uso de tecnologias móveis na assistência à saúde, de forma a aperfeiçoar a comunicação entre profissional e usuário, bem como a autogestão de determinadas condições clínicas^(4,5)^. Avanços tecnológicos atrelados à maior disseminação de recursos digitais no mercado e acessibilidade pela população em geral têm favorecido a inserção dessas tecnologias no âmbito da saúde, tanto na assistência quanto no gerenciamento do cuidado^(4)^. Nesse contexto, diversos aplicativos *mHealth* estão disponíveis em lojas virtuais, com o objetivo de ampliar as possibilidades terapêuticas em diferentes áreas. Entretanto, o rigor científico na estruturação dos conteúdos e o emprego de requisitos mínimos de qualidade da engenharia de *softwares* para o desenvolvimento dessas tecnologias ainda são incipientes^(6)^.

Metodologias da engenharia de *software* visam estabelecer requisitos de qualidade aos aplicativos *mHealth* ^(7)^, com destaque para a *International Organization for Standardization*/*International Electrotechnical Commission* (ISO/IEC) 25010:2011 *System and software engineering – Systems and software Quality Requirements and Evaluation* (SQuaRE)^(8)^, que define parâmetros para especificar, medir e avaliar *softwares* nos quesitos da qualidade de produto e de uso. Portanto, o desenvolvimento de uma tecnologia com garantia de requisitos de qualidade pode impactar significativamente o alcance de melhores resultados clínicos do usuário.

Em uma busca sistematizada realizada nas plataformas *Play Store* ^®^ e *App Store* ^®^, com o objetivo de avaliar a adequação e utilidade de aplicativos móveis direcionados ao tratamento conservador da IU masculina, foram identificados apenas três *softwares* ^(9)^. Na avaliação, foram encontradas restrições quanto ao idioma, à funcionalidade e aos recursos oferecidos, especialmente no que tange às orientações sobre como executar o tratamento e ausência de suporte técnico aos usuários^(9)^. Assim, na perspectiva de propor uma nova tecnologia digital com vistas ao autocuidado e à melhoria da qualidade de vida do público masculino, este estudo teve como objetivo descrever as etapas de desenvolvimento e análise de qualidade de um aplicativo *mHealth* para tratamento da IU em homens pós-prostatectomia radical.

## MÉTODO

### Desenho e Local do Estudo

Trata-se de estudo de desenvolvimento e avaliação tecnológica que visa à construção de um aplicativo *mHealth* baseado na metodologia de processo unificado^(7)^. A pesquisa foi realizada simultaneamente em duas instituições públicas de ensino superior do Brasil, sendo uma localizada em Minas Gerais e outra em Goiás. A condução ocorreu em duas fases, desenvolvimento e análise da qualidade de produto^(8)^, no período de abril de 2021 a março de 2022.

### Critérios de Seleção e Definição da Amostra

Foi considerada uma amostra de 16 especialistas para a fase de análise da qualidade de produto^(10,11)^, sendo oito com experiência na área clínica relacionada à IU e oito na área de desenvolvimento de *softwares*. O levantamento de potenciais especialistas ocorreu por meio de amostragem por conveniência do tipo bola de neve^(12)^. Foi solicitada aos membros dos grupos de pesquisa a indicação de profissionais com experiência nas áreas, na perspectiva de obter maior acurácia à avaliação.

Os critérios utilizados para seleção dos especialistas foram adaptados de um sistema de pontuação para estudos de validação^(13)^. Consideraram-se os seguintes critérios: possuir tese ou dissertação na temática disfunções miccionais ou desenvolvimento de *software* (5 pontos/título); enfermeiros com especialização em estomaterapia, urologia ou disfunções do assoalho pélvico e profissional da ciência da computação especialista em desenvolvimento de *software* (2 pontos/título); participação em grupos ou projetos de pesquisa relacionados à assistência de enfermagem nas disfunções miccionais ou desenvolvimento de *software* (1 ponto); experiência docente na temática disfunções miccionais ou desenvolvimento de *software* (1 ponto/ano); autoria em pelo menos três trabalhos relacionados a disfunções miccionais ou desenvolvimento de *software* publicados em periódicos nos últimos cinco anos (0,5 ponto/trabalho); participação em banca avaliadora de trabalhos com a temática disfunções miccionais ou desenvolvimento de *software* (0,5 ponto/trabalho)^(13)^. Foram incluídos profissionais que atingiram pontuação mínima de cinco pontos^(13)^ e excluídos aqueles que não tinham acesso a dispositivos com sistema operacional *Android*, devido à impossibilidade de acessar o ambiente de simulação.

Os especialistas selecionados foram convidados via *e-mail*. Todos os participantes receberam um guia de instruções para acesso ao *software* e um *link* para preenchimento do formulário de avaliação. Os especialistas em desenvolvimento de *software* receberam também o relatório técnico do aplicativo *mHealth* com a descrição de todo o fluxo de desenvolvimento, recursos empregados e funcionalidades.

### Coleta de Dados

O desenvolvimento do aplicativo, guiado pelo referencial de processo unificado^(7)^, compreendeu cinco etapas: comunicação (levantamento de requisitos, definição dos objetivos e funções do *software*, seleção de recursos materiais); planejamento (descrição das tarefas, levantamento dos riscos prováveis e definição de cronograma de trabalho); modelagem (refinamento do projeto para compreender as necessidades do *software*); construção (elaboração do projeto); e entrega (finalização do *software* e avaliação do produto).

Na etapa comunicação, foi conduzido um estudo descritivo, transversal para identificar aplicativos disponíveis para *download* nas lojas virtuais *App Store* ^®^ e *Play Store* ^®^, direcionados ao público masculino com IU^(9)^. Foram analisados 22 aplicativos por meio de um instrumento adaptado, conforme abrangência do aplicativo, literatura utilizada, compras no aplicativo, conectividade, anúncios, campo de pesquisa de texto, compatibilidade entre dispositivos, imagens/figuras, vídeos e recursos especiais. Constatou-se que nenhum aplicativo alcançou a pontuação máxima desejada. As principais lacunas de conteúdo clínico e de funcionalidades identificadas nos aplicativos disponíveis foram informações sucintas sobre os exercícios, falta de embasamento teórico científico dos conteúdos apresentados, e ausência de imagens ou vídeos relacionados à IU ou ao aparelho urinário para exemplificar como os exercícios deveriam ser realizados ou para auxiliar a localização dos músculos da pelve. Tais lacunas reforçaram a necessidade de desenvolvimento de um novo *software* com maior especificidade para o público-alvo.

A etapa subsequente, denominada planejamento, compreendeu a construção do projeto do *software*, por meio de reuniões remotas de periodicidade quinzenal. Foram definidos os ambientes de desenvolvimento do aplicativo com opção por uso de plataformas e ferramentas gratuitas. A loja virtual *Play Store* ^®^ foi considerada para disponibilizar o aplicativo, devido à sua abrangência^(14)^ e por demandar menos recursos financeiros para a manutenção do *software* na plataforma.

Na etapa de modelagem, foi estruturada a interface do aplicativo *mHealth* a partir do conteúdo e imagens da tecnologia educacional impressa intitulada “Manual de orientações sobre incontinência urinária pós-prostatectomia radical”^(8,15)^ e diretrizes clínicas da *International Continence Society* (ICS)^(2)^. Os conteúdos versaram sobre realização da cirurgia de prostatectomia radical e suas complicações, definição de IU e seu tratamento, orientações sobre os exercícios para treinamento dos músculos do assoalho pélvico, diário vesical e hábitos comportamentais que auxiliam no controle da IU. Para definição do nome do aplicativo, foi realizada consulta a uma amostra não probabilística do tipo intencional, composta por sete profissionais médicos e 12 enfermeiros que atuam na área da urologia e disfunções miccionais. Foi disponibilizado um formulário na plataforma *Google Forms* ^®^ que contemplava o objetivo do aplicativo, o público-alvo e o conteúdo a ser disponibilizado na tecnologia, bem como quatro opções de nomes para votação. Nessa etapa, também foram estabelecidas estratégias de acessibilidade^(16)^, a serem empregadas no desenvolvimento do aplicativo, como uso de cores neutras, ícones de fácil compreensão, linguagem simples com frases curtas, simplicidade de comandos para minimizar falhas de acesso, animações para confirmação de comando entre telas, orientações em áudio, sinais sonoros e vibração do dispositivo para mensagens de alerta.

A etapa de construção baseou-se na produção do aplicativo *mHealth* com codificação da tecnologia em linguagem computacional e armazenamento na *Play Store* ^®^. A interface foi desenvolvida na linguagem de programação *Javascript* com o *framework React Native*. Para armazenamento das informações, foi utilizado o banco de dados MySql. A ferramenta Figma também foi considerada para configuração e diagramação das telas, imagens, botões, fontes e cores. A plataforma Expo^®^ foi utilizada para simular o ambiente de produção durante a fase de homologação de *softwares* e garantir acesso de todos os pesquisadores ao aplicativo *mHealth* em dispositivos com sistema operacional *Android*.

A última etapa, denominada entrega, compreendeu a navegação no aplicativo por seis pesquisadores, a fim de checar o funcionamento de toda a interface e o registro de falhas. Posteriormente, a versão corrigida do *software* foi disponibilizada na plataforma Expo^®^ para análise de qualidade de produto por especialistas. A análise de qualidade do aplicativo *mHealth* seguiu as determinações do modelo de qualidade de produto^(8)^. Esse modelo avalia oito características de qualidade, tais como adequação funcional, eficiência de desempenho, compatibilidade, usabilidade, confiabilidade, segurança, manutenibilidade e portabilidade. Cada uma dessas características apresenta suas respectivas subcaracterísticas (n = 22), conforme apresentado na Figura 1.

**Figura 1:** Características e subcaracterísticas do modelo de qualidade de produto de *software* utilizado para avaliação do aplicativo mHealth IUProst^®^.

Fonte: adaptada da ISO/IEC 25010^(8)^.

Os formulários de avaliação foram adaptados a partir de dois instrumentos disponíveis na literatura^(10,17)^ e estruturados no *Google Forms* ^®^. O formulário dos especialistas da área clínica contemplou 24 itens, que avaliaram quatro características: adequação funcional; usabilidade; confiabilidade; e segurança. Já o formulário dos especialistas em desenvolvimento de *software* contemplou 33 itens, que avaliaram seis características: adequação funcional; eficiência de desempenho; compatibilidade; usabilidade; confiabilidade; e segurança^(8)^. As características manutenibilidade e portabilidade foram desconsideradas para os dois grupos de especialistas, visto que não era possível fornecer o código fonte do *software* aos participantes^(8)^ (Figura 1).

Para cada subcaracterística avaliada (Figura 1), foi atribuída uma classificação/pontuação: concordo (1 ponto); não concordo (zero ponto); não se aplica (zero ponto). Para respostas “não concordo”, o especialista poderia justificar e sugerir modificações no campo de comentários. Foi estabelecido um prazo máximo de 30 dias para retorno dos participantes. Os pesquisadores poderiam ser acionados pelo *WhatsApp* ^®^, *e-mail* ou ligações telefônicas, para solucionar possíveis problemas técnicos de acesso ao aplicativo.

### Análise e Tratamento dos Dados

Os dados foram transcritos para planilhas do *Microsoft Excel* ^®^, e os valores em percentuais das características de qualidade foram obtidos a partir da fórmula adaptada: Vc = ∑ Vsca /(a + d + na – na) × 100^(16,17)^, em que: valor medido da característica (Vc) é igual ao somatório dos valores das subcaracterísticas com respostas concordo (∑Vsca), dividido pela soma de respostas concordo (a), não concordo (d) e não se aplica (na), subtraído pelas respostas não se aplica (na), multiplicado por 100.

Para o cálculo da média e desvio padrão dos valores das subcaracterísticas, foi excluída a resposta “não se aplica”, por se referir a questões que os especialistas não tinham capacidade de avaliar, seja por falta de recursos técnicos ou por especificidade de conhecimento. Tal resposta não pontuava e, portanto, não comprometeu a avaliação^(17)^.

Para análise dos resultados, foram considerados como satisfatórias as características que obtiveram o percentual de concordância superior a 70%, conforme a escala de avaliação para subcaracterísticas^(10,18)^, sendo 25% (fraco), 50% (regular), 75% (bom) e 100% (excelente). Todas as sugestões feitas pelos especialistas no campo “comentários” foram analisadas pelos pesquisadores quanto à pertinência e viabilidade para prosseguir com os ajustes técnicos e estruturais do *software*. Sugestões que implicavam gastos financeiros e execução em tempo não aplicável ao cronograma estabelecido foram consideradas para futuras atualizações.

### Aspectos Éticos

A pesquisa foi aprovada no Comitê de Ética em Pesquisa da Universidade Federal de Minas Gerais, sob Parecer nº 4.864.981 e CAAE nº 41736921.5.0000.5149, em consonância com as recomendações da Resolução nº 466/12 do Conselho Nacional de Saúde. Os voluntários que participaram das cenas dos vídeos que compõem o aplicativo assinaram o Termo de Autorização de Uso da Imagem. A locutora dos vídeos é uma das pesquisadoras principais, que assinou o Termo de Autorização do Uso de Voz. Elaborou-se também o Termo de Consentimento Livre e Esclarecido (TCLE), disponível no aplicativo para autorização de cada usuário sobre uso de dados clínicos. Os princípios estabelecidos na Lei Geral de Proteção de Dados também foram considerados durante a navegação no aplicativo, a fim de garantir a integridade e o cuidado à privacidade dos dados de cada usuário.

Após a avaliação da qualidade do produto, o aplicativo foi homologado e disponibilizado para *download* na *Play Store* ^®^. O registro do aplicativo IUProst^®^ foi aprovado pelo Instituto Nacional da Propriedade Industrial (INPI), com validade de 50 anos a partir de 1º de janeiro subsequente à data de 10/03/2022, conforme o Artigo 2º da Lei 9.609, de 19 de fevereiro de 1998, Processo nº BR512022001279-0.

## RESULTADOS

O nome selecionado para o aplicativo foi “IUProst^®^”, que representa a junção da sigla “IU”, de incontinência urinária, e “Prost”, enquanto radical das palavras próstata e prostatectomia. A logomarca contempla uma mão na cor azul claro, que expressa o sentido de ajuda, proteção e cuidado; uma gota ao centro na cor azul escuro, que remete à perda urinária; e um elemento ao centro da gota na cor branca, que representa o homem (Figura 2).

**Figura 2:** Logomarca do aplicativo IUProst^®^ (2022).

Para acesso inicial ao aplicativo, foram elaboradas quatro telas de abertura com informações breves sobre o propósito do aplicativo, TCLE, cadastro, *login* e personalização. A tela de personalização do usuário refere-se à inserção de dados clínicos e ao preenchimento de dois instrumentos clínicos: o *International Consultation on Incontinence Questionnaire – Short Form* (ICIQ-SF)^(19)^; e a Escala de Incontinência Urinária Pós-Prostatectomia Radical (EIUPR)^(20)^.

Em seguida, o usuário é direcionado a um menu principal que contempla oito telas. Na tela “Sobre o IUProst^®^”, há informações gerais sobre o aplicativo com dados do grupo de pesquisa e referências que sustentam o conteúdo apresentado.

A tela “Meus exercícios” foi desenvolvida para orientar o usuário acerca da realização do treinamento dos músculos do assoalho pélvico. Os exercícios propostos foram organizados em oito etapas, a serem executadas ao longo de oito semanas (dois meses), nos períodos da manhã, tarde e noite. Para facilitar a compreensão sobre como realizar os exercícios, o usuário pode acessar breves explicações em formato de texto, áudio e vídeo.

A tela “Meu diário vesical” apresenta o cálculo da quantidade de líquidos recomendada para ingestão diária^(21)^, um espaço para registro de tipo, volume e horário de líquidos ingeridos, frequência de eliminações e perdas urinárias, frequência de trocas de forros, absorventes ou fraldas.

A tela “Meu tratamento” contém informações referentes à anatomia do sistema urinário masculino, o que é a cirurgia de prostatectomia radical, reconhecimento dos músculos do assoalho pélvico, além de dicas sobre hábitos de vida que favorecem a melhora da IU. Entrevistas realizadas com dois usuários com IU pós-prostatectomia radical e um familiar com relatos de experiências e desafios enfrentados diante do diagnóstico inicial e tratamento pós-cirúrgico estão disponíveis em uma tela denominada “Experiência do paciente”.

Em “Minhas conquistas”, o usuário acompanha o seu desempenho no tratamento por meio de um gráfico que representa a execução dos exercícios nos últimos sete dias e o consumo diário de líquidos. Já a tela “Fale com o profissional” viabiliza o contato entre usuários e profissionais de enfermagem com experiência na área da urologia por e-mail e pelas redes sociais *WhatsApp* ^®^ e *Instagram* ^®^. O público, portanto, pode esclarecer dúvidas e acessar informações relacionadas à prevenção, tratamento e hábitos de vida saudáveis.

Em “Configurações e ajustes”, o usuário configura o aplicativo conforme suas preferências, com programação de horários e alertas sonoros para lembrá-lo de realizar os exercícios de treinamento dos músculos do assoalho pélvico. Algumas telas que compõem o aplicativo IUProst^®^ estão apresentadas na Figura 3.

**Figura 3:** Telas do menu principal do aplicativo IUProst^®^. Belo Horizonte, MG, Brasil (2022).

No que concerne à avaliação da qualidade do produto^(8)^, 30 avaliadores foram convidados para participar da pesquisa: 15 enfermeiros docentes e/ou especialistas na área e 15 especialistas em desenvolvimento de *software*. Desses, apenas oito enfermeiros e oito especialistas em desenvolvimento de *software* responderam previamente ao convite. Dos oito especialistas da área clínica, a média de idade foi de 44,6 anos (± 6,1) e tempo médio de formação de 19,7 anos (± 6,0). Todos possuem experiência docente na temática, e 87,5% atuam na prática clínica. Quanto aos oito especialistas em desenvolvimento de *software*, a média de idade foi de 36,7 anos (± 8,9), e o tempo médio de formação, de 8,8 anos (± 9,1). A maioria dos desenvolvedores (75,0%) também possui experiência docente em tecnologia da informação, e 87,5% possuem atuação prática na área. Quanto à localização geográfica, os participantes residiam nas regiões Centro-Oeste (n = 09), Nordeste (n = 03), Sudeste (n = 03) e Sul (n = 01). Conforme os critérios de seleção^(13)^, especialistas da área clínica obtiveram pontuação mínima de 21,0 e máxima de 42,5 pontos, enquanto que especialistas em desenvolvimento de *software* obtiveram pontuação mínima de 12,0 e máxima de 54,0 pontos.

Todas as características analisadas quanto à qualidade do produto apresentaram índices superiores a 70%. Na análise das 22 subcategorias, duas obtiveram concordância fraca (acessibilidade e proteção contra erros do usuário). Outras duas subcategorias apresentaram concordância regular (maturidade e recuperabilidade). Os índices de concordância entre os especialistas da área clínica e de desenvolvimento de *software* estão descritos na Tabela 1. Ressalta-se que especialistas da área clínica não avaliaram as características e subcaracterísticas da eficiência de desempenho, compatibilidade, não repúdio e responsabilização, por se tratarem de tópicos mais específicos da área da ciência da computação.

**Tabela 1:** Índice de concordância na avaliação da qualidade do produto entre especialistas da área clínica e de desenvolvimento de *software* – Belo Horizonte, MG, Brasil, 2022.

Característica e subcaracterísticas	Especialistas da área de desenvolvimento de *software* Média (DP)	Especialistas da área clínica Média (DP)
**Adequação funcional**	**100,00 (0,0)**	**78,5 (7,1)**
Completude funcional	100,0 (0,0)	85,7 (0,0)
Correção funcional	100,0 (0,0)	78,5 (10,1)
Adequação funcional	100,0 (0,0)	71,4 (0,0)
**Eficiência de desempenho**	**90,5 (8,4)**	**–**
Comportamento em relação ao tempo	87,5 (0,0)	**–**
Utilização de recursos	100,0 (0,0)	**–**
Capacidade	84,0 (21,9)	**–**
**Compatibilidade**	**100,0 (0,0)**	**–**
Interoperabilidade	100,0 (0,0)	**–**
**Usabilidade**	**82,7 (18,9)**	**74,2 (24,0)**
Reconhecimento de adequação	96,8 (6,2)	92,2 (9,0)
Apreensibilidade	91,6 (14,4)	85,7 (0,0)
Operabilidade	86,6 (1,3)	77,3 (8,4)
Acessibilidade	50,0 (0,0)	40,0 (0,0)
Proteção contra erros do usuário	71,4 (0,0)	50,0 (0,0)
Estética de interface com o usuário	100,0 (0,0)	100,0 (0,0)
**Confiabilidade**	**82,3 (20,5)**	**95,0 (10,0)**
Maturidade	62,5 (0,0)	80,0 (0,0)
Tolerância a falhas	**100,0 (0,0)**	**100,0 (0,0)**
Recuperabilidade	66,6 (0,0)	100,0 (0,0)
Disponibilidade	100,0 (0,0)	100,0 (0,0)
**Segurança**	**91,4 (9,3)**	**87,6 (5,4)**
Confidencialidade	100,0 (0,0)	85,7 (0,0)
Integridade	93,7 (8,8)	93,7 (8,8)
Não repúdio	100,0 (0,0)	**–**
Responsabilização	83,3 (0,0)	**–**
Autenticação	80,0 (0,0)	83,3 (0,0)

Nota: DP – desvio padrão.

No Quadro 1, são apresentados os comentários dos especialistas que implicaram melhorias técnicas e estruturais do *software*.

**Quadro 1:** Sugestões dos especialistas da área clínica e de desenvolvimento de *software* atendidas na versão 1 do aplicativo IUProst^®^ – Belo Horizonte, MG, Brasil, 2022 (n = 16).

Característica de qualidade	Especialistas da área de desenvolvimento de *software*	Especialistas da área clínica
**Adequação funcional**	– Corrigir evolução de progresso durante o lançamento de líquidos ingeridos, volume de urina e de exercícios realizados (Especialista 8). – Corrigir ativação do áudio de um dos vídeos (Especialistas 1 e 2).	– Especificar mudanças nos hábitos de vida de acordo com a queixa do paciente (Especialista 2). – Adicionar informação de como relaxar a musculatura pélvica (Especialista 2).
**Eficiência de desempenho**	– Informar a capacidade de armazenamento do banco de dados (Especialista 2). – Melhorar o tempo de carregamento do aplicativo (Especialista 4).	(Não se aplica a esse grupo de especialistas).
**Compatibilidade**	Não houve comentários/sugestões.	(Não se aplica a esse grupo de especialistas).
**Usabilidade**	– Inserir campos de resposta único, evitando campos para resposta aberta (Especialista 3). – Inserir “combos” em cadastro do estado e cidade (Especialista 7). – Modificar campos dos formulários para não permitir inserção de dados com valores absurdos e inválidos (Especialista 6). – Incluir na seção “Meus exercícios” uma tela com explicação da etapa (Especialista 8). – Inserir a opção de aumentar o tamanho da fonte dos textos (Especialista 7).	– Incluir em dados clínicos a opção de desmarcar seleção em doenças prévias (Especialista 7). – Inserir a opção de inexistência de doenças prévias (Especialista 3). – Permitir que, na primeira questão do questionário de qualidade de vida, seja possível preencher mais de uma alternativa (Especialista 4). – Fornecer suporte de acessibilidade para pessoas com deficiência, tais como comandos verbais para as perguntas dos questionários e das informações sobre IU (Especialista 8). – Ajustar a linguagem utilizada no aplicativo, de forma a torná-la mais acessível ao usuário (Especialista 1). – Inserir etapas obrigatórias ou enumerar as etapas de tratamento para que o usuário saiba por qual deve começar (Especialista 3).
**Confiabilidade**	– Inserir registro de progresso após entradas de dados (Especialista 1).	Não houve comentários/sugestões.
**Segurança**	– Inserir avaliação de força da senha (Especialista 2). – Incluir um mínimo de caracteres para a criação de senhas (Especialista 1).	– Inserir mecanismo de recuperação de senha (Especialista 4). – Inserir a opção “entrar automaticamente/salvar senha neste dispositivo” (Especialista 4).

## DISCUSSÃO

O IUProst^®^ foi desenvolvido como uma tecnologia *mHealth* capaz de auxiliar profissionais de saúde no acompanhamento e monitoramento do tratamento conservador para a IU pós-prostatectomia radical. Essa ferramenta tecnológica se destaca por contemplar conteúdos específicos para o público masculino no que concerne à orientação clínica sobre IU, na perspectiva de aumento da adesão ao tratamento por meio de estratégias interativas, dinâmicas e ajustáveis à rotina do usuário. A expectativa da tecnologia também é fortalecer a relação usuário-profissional, ao viabilizar canais de comunicação e favorecer a capacidade de autogestão do cuidado.

As tecnologias de informação e comunicação relacionadas à saúde são um conjunto de ferramentas que contribuem para o aprimoramento das informações de saúde e apoio diagnóstico. Podem oferecer facilidade na transmissão de informações por meio digital, ao ultrapassar obstáculos como distância, ampliar a atenção, a cobertura de cuidados, bem como reduzir custos^(22,23)^. Nesse contexto, os aplicativos *mHealth* têm se tornado relevantes para a assistência à saúde, por se apresentarem como ferramentas que otimizam e simplificam o processo terapêutico, além de contribuírem com a ampliação do acesso à informação e o maior engajamento do usuário ao tratamento^(24)^. Com o avanço dos recursos tecnológicos, espera-se que essas ferramentas sejam incorporadas gradativamente à prática clínica, com vistas a auxiliar no tratamento e reabilitação de determinadas condições clínicas^(5)^, bem como promover a educação em saúde^(25)^.

No contexto da IU, no Brasil, a maioria dos aplicativos apresenta qualidade reduzida e funcionalidades limitadas^(9)^, sobretudo devido à escassez de informações pautadas em bases científicas confiáveis^(26)^. Neste sentido, o IUProst^®^ foi desenvolvido a partir das principais diretrizes clínicas da ICS^(2)^, estruturado conforme o conteúdo de um programa cognitivo comportamental para tratamento da IU pós-prostatectomia radical com efetividade clínica comprovada em ensaios clínicos^(8,15)^. Além disso, o aplicativo considera as singularidades dos usuários, por contemplar funcionalidades que favorecem o acompanhamento da sua evolução clínica e a personalização de comandos, conforme suas preferências e necessidades de uso.

Pesquisadores^(26)^ afirmam que, apesar do grande número de aplicativos desenvolvidos com a finalidade de apoiar pacientes com IU, ainda são escassos aplicativos centrados nas necessidades do paciente como forma de promover melhores experiências e resultados. O tratamento conservador, enquanto primeira escolha terapêutica para a IU, abrange o treinamento da musculatura do assoalho pélvico em associação a mudanças de hábitos de vida^(2)^. Por envolver o comportamento humano, requer estratégias e ferramentas que promovam reforço das informações durante o processo terapêutico, a fim de aumentar a adesão ao tratamento e a efetividade clínica. Portanto, torna-se indispensável o uso de tecnologias válidas e de qualidade que auxiliem profissionais e usuários nesse propósito de mudança comportamental^(27)^.

Analisar a qualidade dos aplicativos *mHealth* é fundamental para a proposição de melhorias que favoreçam a navegabilidade, a acessibilidade digital e, consequentemente, maior adesão à tecnologia^(27)^. A qualidade efetiva de um *software* é considerada quando a ferramenta dispõe de elementos que a torne útil e que forneça valor mensurável, em diversos cenários, com foco para os desenvolvedores e para os usuários^(28,29)^. Assim, é possível propor um produto que atenda às necessidades do público-alvo^(2)^. Nessa perspectiva, o IUProst^®^ foi avaliado por dois grupos de especialistas com *expertises* diferentes, o que permitiu maior robustez ao processo de análise de qualidade^(8)^.

O IUProst^®^ obteve índices de concordância satisfatórios para todas as características avaliadas^(8)^, o que favorece a sua recomendação como uma ferramenta tecnológica aliada ao tratamento, embora não substitua o atendimento profissional e presencial. A característica adequação funcional obteve concordância de 100% entre os desenvolvedores. No entanto, entre os especialistas da área clínica, essa característica apresentou concordância de 78,5%. Esse resultado pode estar associado às sugestões de melhoria do conteúdo clínico do aplicativo, tais como especificar as mudanças nos hábitos de vida de acordo com a queixa do paciente e adicionar informação sobre como realizar o relaxamento da musculatura pélvica.

Sobre informações acerca do relaxamento da musculatura pélvica, ressalta-se que, na aba “Meu tratamento”, o usuário tem acesso a orientações sobre como reconhecer os seus músculos e como relaxar e contrair corretamente essa musculatura, além dos vídeos explicativos presentes em cada semana de treinamento. Portanto, a baixa concordância da característica adequação funcional entre os especialistas da área clínica pode estar associada ao fato de que alguns avaliadores não tenham acessado todos os recursos disponíveis na aplicação, o que repercutiu em um resultado inferior ao esperado na avaliação dessa característica.

Sobre a confiabilidade, duas subcaracterísticas obtiveram uma concordância regular pelos desenvolvedores, como maturidade (62,5%) e recuperabilidade (66,6%). A maturidade diz repeito às falhas técnicas, e a recuperabilidade, à capacidade de recuperar dados inseridos após mensagem de erro^(8)^. De fato, um avaliador relatou que não houve registro de progresso da etapa do exercício. Contudo, para solucionar essa demanda, foi realizada uma correção no fluxo de gravação dos registros do progresso dos exercícios, sem prejuízos subsequentes.

Apesar de a característica de usabilidade ter obtido índices de concordância satisfatórios entre as duas especialidades, a subcaracterística acessibilidade apresentou concordância fraca entre especialistas da área clínica e densenvolvedores. A acessibilidade diz respeito ao suporte de pessoas com deficiência, e, a partir dos resultados obtidos, considera-se que esse requisito necessita de melhorias no IUProst^®^. Para aprimorar esse requisito, especialistas da área clínica sugeriram a inclusão de comandos verbais na tela que contempla os instrumentos de avaliação do usuário. Contudo, é importante destacar que a acessibilidade auditiva não foi planejada pelos desenvolvedores nesta primeira versão do *software*. Em contrapartida, ressalta-se a inclusão de áudios e vídeos nas telas relacionadas à execução dos exercícios de treinamento muscular, o que favorece o uso do aplicativo por homens com deficiência visual.

Outra subcaracterística de usabilidade que apresentou concordância fraca entre especialistas da área clínica foi a proteção contra erros do usuário. Nesse requisito, o objetivo foi investigar se há informação ao usuário em casos de entrada de dados inválidos^(8)^. Para isso, foi proposta a inserção de campos de resposta únicos que substituíram todos os campos para resposta aberta.

Na avaliação da eficiência de desempenho, que foi realizada apenas pelos especialistas desenvolvedores, as subcaracterísticas comportamento em relação ao tempo, utilização de recursos e capacidade obtiveram índices de concordância bom, excelente e bom, respectivamente. Esse resultado pode ser justificado pelo fato de o IUProst^®^ possuir um servidor exclusivo para armazenamento de dados. A utilização desse servidor também contribui para a construção de um banco de dados clínicos, o que subsidia a realização de futuras pesquisas epidemiológicas, bem como a implementação de *dashboards* para acompanhamento clínico dos usuários por profissionais de saúde.

Apesar de o IUProst^®^ ter sido desenvolvido para servir de apoio ao tratamento de homens com IU pós-prostatectomia radical, o *software* também pode ser útil no processo educacional do profissional. Além do protocolo de exercícios, o programa cognitivo comportamental contém orientações sobre os principais hábitos de vida, além de explicações sobre anatomia, fisiologia, ensinando sobre o preenchimento adequado do diário vesical. Esse diário refere-se a uma ferramenta preconizada pela ICS^(2)^ de baixo custo, que permite caracterizar o hábito miccional diário do indivíduo e propor melhores intervenções para a reabilitação da eliminação urinária.

Como limitações do estudo, destacam-se a incompatibilidade do aplicativo com outros sistemas operacionais, o que dificulta a avaliação do *software* por especialistas que não possuem dispositivos móveis *Android*, bem como a não realização da avaliação da qualidade de uso por homens que vivenciam a condição de saúde. Portanto, a equipe de pesquisa tem como propósitos de curto prazo a disponilização do IUProst^®^ no sistema operacional iOS e a avaliação pelo público-alvo por meio de estudos de análise de efetividade clínica. A equipe de pesquisa também avançará no *redesign* do *software*, conforme as boas práticas para o letramento digital em saúde^(30)^, na perspectiva de inclusão de novos elementos que facilitem a navegação, a compreensão e o emprego clínico das informações de saúde pelo usuário.

## CONCLUSÃO

O desenvolvimento do IUProst^®^ alcançou os requisitos previstos nas etapas de comunicação, planejamento, modelagem, construção e entrega. Constatou-se que todas as características de qualidade avaliadas no IUProst^®^ foram satisfatórias, o que demonstra que o *software* apresenta qualidade técnica e clínica para atender às necessidades de homens com IU após tratamento cirúrgico para o câncer de próstata.

O IUProst^®^ é uma inovação tecnológica em saúde que favorece a atuação do enfermeiro no cuidado a homens com IU pós-prostatectomia radical, a partir de informações válidas acerca da IU masculina. A tecnologia permite ao usuário acesso a orientações quanto a mudanças de hábitos de vida e realização de exercícios para fortalecimento dos músculos do assoalho pélvico, de forma remota e a qualquer momento, por meio de um *smartphone*.
